# Cathepsin B prevents cell death by fragmentation and destruction of pathological amyloid fibrils

**DOI:** 10.1038/s41420-025-02343-w

**Published:** 2025-02-15

**Authors:** Maksim I. Sulatsky, Olesya V. Stepanenko, Olga V. Stepanenko, Ekaterina V. Mikhailova, Anna I. Sulatskaya

**Affiliations:** 1 Laboratory of cell morphology, Institute of Cytology Russian Academy of Sciences, St. Petersburg, Russia; 2 Laboratory of structural dynamics, stability and folding of proteins, Institute of Cytology Russian Academy of Sciences, St. Petersburg, Russia

**Keywords:** Cell death, Protein aggregation, Protein aggregation, Proteolysis

## Abstract

Amyloid fibrils cause organ and tissue dysfunction in numerous severe diseases. Despite the prevalence and severity of amyloidoses, there is still no effective and safe anti-amyloid therapy. This study investigates the impact of cysteine protease cathepsin B (CTSB) on amyloids associated with Alzheimer’s and Parkinson’s diseases, hemodialysis, and lysozyme amyloidosis. We analyzed the effect of CTSB on the size, structure, and proteotoxicity of amyloid fibrils formed from alpha-synuclein, abeta peptide (1-42), insulin, and lysozyme using a combination of spectroscopic, microscopic, electrophoretic, and colorimetric methods. Our comprehensive research revealed a dual effect of CTSB on amyloid fibrils. Firstly, CTSB induced amyloid fragmentation while preserving their ordered morphology, and, secondly, it “loosened” the tertiary structure of amyloids and reduced the regularity of the secondary structure. This dual mechanism of action was universal across fibrils associated with different pathologies, although the disruption efficacy and predominant type of degradation products depended on the amyloids’ structure, size, and clustering. Notably, CTSB-induced irreversible degradation significantly reduced the toxicity for immortalized and primary cell lines of low-clustered fibrils, such as alpha-synuclein amyloids associated with Parkinson’s disease. These findings enhance our understanding of how endogenous CTSB may regulate amyloid content at the molecular level in different neuropathologies. In addition, our results suggest the potential of CTSB as a component of anti-amyloid drugs in combination with agents that enhance the accessibility of proteolytic sites within amyloid clots and reduce these clusters stability.

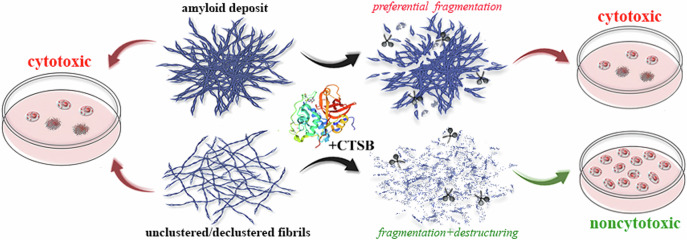

## Introduction

Diseases accompanied by the accumulation of ordered protein aggregates, amyloid fibrils, are becoming increasingly common each year. Specifically, about 6.1 million patients suffer from Parkinson’s disease [1–3], a neurodegenerative condition marked by the formation of pathogenic amyloids from alpha-synuclein. Alzheimer’s disease, characterized by the accumulation of plaques from abeta peptide (1-42) [4–6] in the brain, is even referred to as the “epidemic of the 21st century” with the World Health Organization predicting it to become the most prevalent disease worldwide in the coming decades [7]. This condition has already been identified in 43.8 million individuals, and this number is expected to rise to 152 million by 2050, according to forecasts [8]. These figures highlight the global scale of the problem associated with the formation of pathological amyloids in the body in several dozen severe diseases [9–14].

Despite the prevalence and severity of amyloidoses, there is still no effective and safe anti-amyloid therapy available [15, 16]. Currently, one of the promising strategies for treating these diseases is the destruction and clearance of mature amyloid fibrils and plaques [17–20]. Several new drugs based on this approach are in various stages of development and clinical trials [21–25]. This strategy is rooted in the “amyloid hypothesis”, a longstanding paradigm that attributes the development of Alzheimer’s disease and other neurodegenerative disorders to the accumulation of amyloid plaques. These insoluble deposits are believed to cause cell death and cognitive impairment characteristic of these diseases. For example, studies have shown that amyloid deposits significantly increase the risk of developing amnestic mild cognitive impairment and its progression to Alzheimer’s disease [26]. Furthermore, cerebral amyloid angiopathy, characterized by plaque accumulation on cerebral vessel walls, interacts with pathological accumulation of neuritic amyloid plaques, exacerbating tau pathology and accelerating cognitive decline [27]. Similarly, in the case of primary or light-chain (AL) amyloidosis, the most common type of systemic amyloidosis, the accumulation of insoluble fibrils in tissues leads to organ dysfunction and, ultimately, to fatal outcomes [28, 29].

However, emerging evidence suggests that fibril formation may not be the sole mechanism driving tissue and cellular damage in amyloidoses. For instance, hyper-aggregation of abeta peptide induced by reduced IGF1 signaling was found to ameliorate Alzheimer’s-like symptoms in model mice, rather than exacerbating them [30]. Moreover, some clinical studies have revealed a weak correlation between amyloid plaque burden and the severity of dementia. For instance, some patients with significant plaque deposition, for some reason, do not exhibit dementia symptoms [31]. These findings highlight additional pathogenic factors beyond plaque accumulation. Among these, oligomeric forms of amyloidogenic proteins have gained attention as potentially more toxic than mature fibrils under certain conditions. For example, abeta peptide dimers were shown to exhibit greater toxicity than large aggregates [32]. Similarly, smaller PrP aggregates demonstrated significantly higher toxicity and infectivity compared to larger fibrils [33].

The accumulating data on the cytotoxicity of various protein species challenge the original “amyloid hypothesis” and expand our understanding of amyloidoses pathogenesis. While insoluble deposits remain the primary pathogenic factor in systemic [28, 29] and some localized amyloidoses [34–37], neurodegenerative diseases associated with amyloids present a more complex scenario. In addition to the direct toxicity of amyloid plaques, these aggregates can also serve as a source from which soluble amyloid forms, including low-molecular-weight oligomers, are released during degradation. These oligomers exhibit comparable, and in some cases even greater, toxicity to neurons [38]. Despite the well-documented toxicity of both mature plaques and these oligomeric intermediates, the development of anti-amyloid drugs often fails to adequately address the analysis of not only the efficiency of mature amyloid clearance but also the cytotoxicity of the low-molecular-weight degradation products.

In this study, we performed such an analysis after cysteine protease cathepsin B (CTSB) treatment, which is being considered as a potential therapeutic agent in several studies [39, 40]. Interest in this enzyme, which participates in the degradation of lysosomal proteins, has arisen because it is expressed in macrophages and multinucleated histiocytic giant cells (MGC) found in close proximity to amyloid deposits in the body [41]. Furthermore, CTSB itself has been detected in the amyloid deposits of patients [41–44]. Given this fact, the question of whether cells of the mononuclear phagocyte system can destroy amyloids either through phagocytosis or by releasing active proteases, including CTSB, is being actively investigated.

In analyzing the potential use of CTSB as a therapeutic agent with neuroprotective and anti-amyloidogenic properties, its ability to prevent the formation of neurotoxic plaques by various amyloidogenic proteins (abeta peptide, immunoglobulin light chain, serum amyloid A, and alpha-synuclein) as well as to cleave amyloid deposits was noted [40–42, 45–50]. It is important to emphasize that the current body of evidence regarding the impact of CTSB on mature amyloids is both limited and highly contradictory. The studies available in the literature, using genetic, immunohistochemical and pharmacological approaches, demonstrated the fundamental ability of the enzyme to degrade amyloids formed from alpha-synuclein and immunoglobulin light chain [40, 41, 50, 51]. Furthermore, the ability of CTSB to reduce the amount of abeta peptide fibrillar structures in vitro was demonstrated, and CTSB over-expression was shown to contribute to the reduction of amyloid deposits formation in aged hAPP mice [42], similar to neprilysin or insulin degrading enzyme (IDE) [52]. Some of these studies suggested that this unique ability of CTSB reveals its potential as a therapeutic agent for diseases where controlling amyloid quantity is a crucial aspect of treatment. However, some studies [48, 51] reported that incomplete degradation of amyloids leads to increased nucleation activity and induction of new aggregate formation, which could potentially exacerbate the disease. Consequently, these authors reached the opposite conclusion that inhibiting the effect of CTSB on mature amyloids might serve as a therapeutic strategy. The consequences of the CTSB gene knockout (or knockdown) in neurologic disorder models was also studied. For example, a number of studies in mouse models of Alzheimer’s disease found improved brain function following knockout of the CTSB gene [53]. Similarly, knockdown of the CTSB gene in the nematode *Caenorhabditis elegans* protected model worms from abeta peptide toxicity [54]. In contrast, the enhanced polyQ-associated proteotoxicity observed upon CTSB knockdown in model worms led to the suggestion that inhibition of CTSB activity should be approached with caution [54].

The aim of current study was to clarify the conflicting views on the effect of CTSB on mature amyloid fibrils by filling the gaps in the understanding of molecular basis of this process and identifying the factors determining the disruption efficiency. For this purpose, we for the first time analyzed in vitro the mechanism of CTSB-induced amyloid degradation, as well as the structure and properties of the resulting degradation products by various biochemical and physicochemical methods including special procedure of sample preparation. To ensure the universality of the observed effects and to identify patterns in the processes observed, we used amyloids formed from several proteins and peptides, significantly differing in structure and properties. Specifically, we examined the degradation of amyloids accumulating in the bodies of patients with Alzheimer’s disease (formed from abeta peptide (1-42)) [55, 56], Parkinson’s disease (formed from alpha-synuclein) [57–59], hemodialysis amyloidosis in acute renal failure (formed from beta-2-microglobulin) [60, 61], and systemic lysozyme amyloidosis (formed from lysozyme) [62, 63].

## Results

### CTSB-induced degradation of model amyloids

**Fig. 1:**
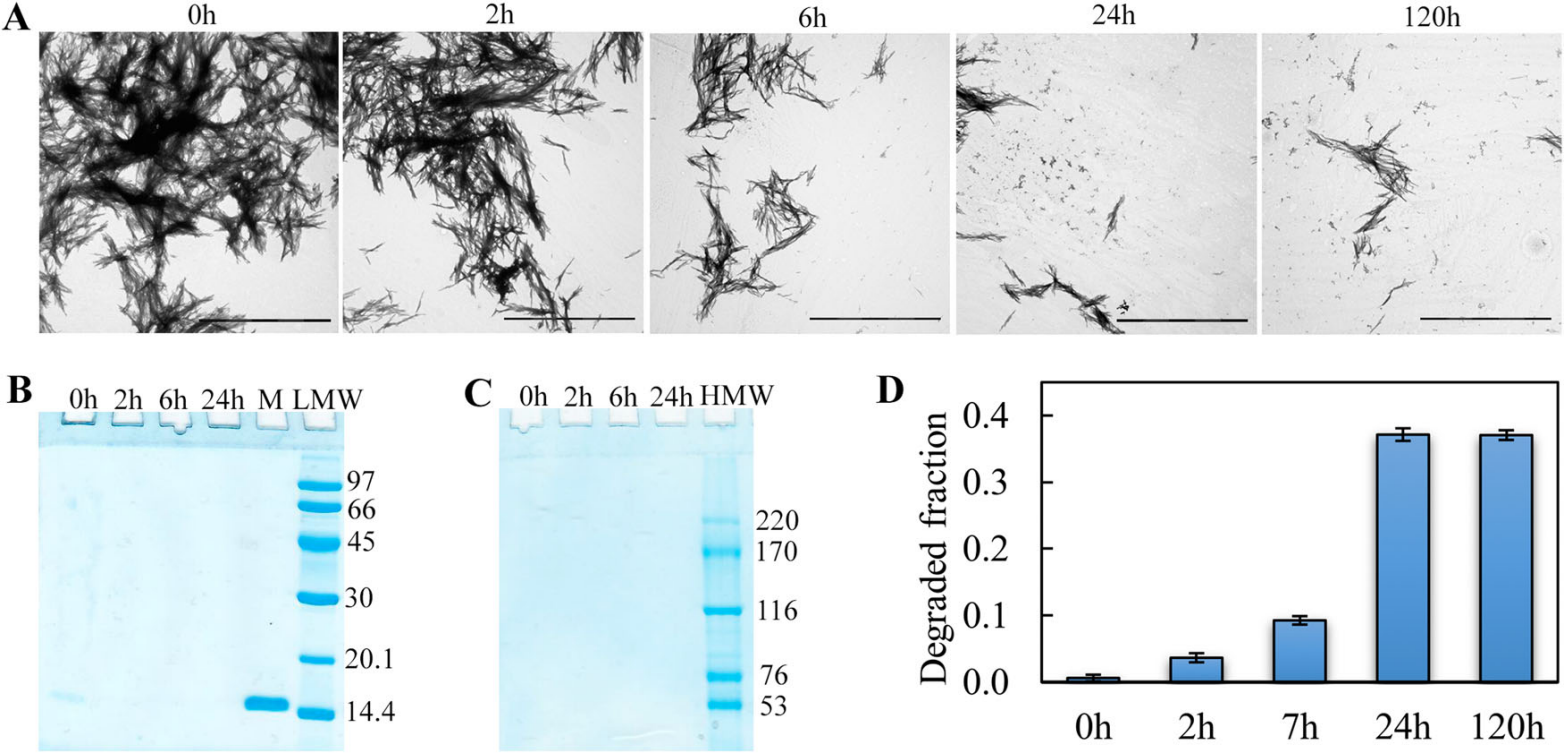
Dynamics of CTSB-induced degradation of lysozyme amyloid fibrils. **A** Visualization of amyloids using transmission electron microscopy before and at different time intervals (2, 6, and 24 h, indicated above the panels) after enzyme addition. Scale bars are 5 μm. Assessment of monomeric/oligomeric protein content in the sample using pseudo-native (**B**) 17% and (**C**) 8% SDS-PAGE. Monomeric protein (M) at a concentration equal to that of amyloids was loaded in lane 5 as a control. Low-molecular-weight (LMW) and high-molecular-weight (HMW) marker proteins were loaded in the last lanes. Their molecular weights are indicated to the right of the gels. **D** Quantification of the degraded fraction in the supernatant using absorption spectroscopy after centrifugation of samples collected at different time intervals (2, 6, 24, and 120 h) after enzyme addition. Values are calculated relative to the concentration of intact amyloid fibrils. Data are mean ± SD (*n* = 5).

To refine research techniques and determine the duration of the experiment, we first analyzed the impact of CTSB on amyloid fibrils formed from lysozyme, which serves as a convenient and accessible model object for studying amyloids in vitro. To visualize the degradation process of mature amyloids and analyze the dynamics of this process, aliquots were collected at 2, 6, and 24 h of incubation (the time after which, according to literature, CTSB completely loses its activity [64]) in the presence of the protease, and then the enzyme was inactivated [65] (see the “Materials and Methods” section). The selected samples were visualized using transmission electron microscopy (TEM) (Fig. 1A). The presence of monomeric or oligomeric protein forms that could not be visualized using TEM was assessed using pseudo-native SDS-PAGE (Fig. 1B, C). The efficiency of amyloid degradation was determined by the proportion of the degraded low-molecular-weight protein fraction using absorption spectroscopy of the supernatants obtained after centrifuging the selected samples (Fig. 1D).

It was shown that under the action of CTSB, a gradual degradation of the studied amyloids occurred (Fig. 1D). We observed a significant reduction in their fiber length (Fig. S1A) and loss of fibrillar morphology of some amyloid fragments 24 h after the start of the experiment (Fig. 1A). We assessed which protein fractions were present in samples before and after CTSB exposure using SDS-PAGE under pseudo-native conditions (Fig. 1B, C) not causing dissociation of aggregates in the sample under study. The sample with intact lysozyme amyloid was found to be virtually free of protein monomer (Fig. 1B, 0 h) of molecular weight 14.3 kDa (monomeric protein at a concentration equal to the amyloid concentration is shown in lane «M» as a control). The intact sample also lacked protein oligomers and small aggregates with molecular weight less than 220 kDa. This confirms that all of the protein in the intact sample is incorporated into substantially larger amyloid fibrils. Given the CTSB-induced degradation of amyloid (Fig. 1А), the appearance of oligomeric fractions of lysozyme and monomeric protein in the samples over time could be expected. However, we did not find an increase in the level of lysozyme monomers or oligomers with a molecular weight of less than 220 kDa in the samples (Fig. 1B, C, lanes «2 h», «6 h», «24 h»), indicating the relatively large size of the degradation products. It was noted that even 24 h after the start of the treatment, amyloid fibrils were still detected in the sample (Fig. 1A).

To ensure that 24 h is sufficient time for the protease’s action on the amyloids and for the system of degradation products to reach equilibrium, we extended the incubation time of the samples to 5 days. It was found that the morphology and size of the fibril degradation products, as well as the proportion of the degraded fraction (which amounted to about 40%), were the same after 24 h and 5 days of protease treatment (Fig. 1A, D). The sufficiency of 24-h exposure for degradation of other tested amyloids was confirmed on the example of amyloid formed from alpha-synuclein (Fig. S2 and S1B). As a result, the duration of the experiments on the degradation of other amyloids by CTSB was limited to 24 h.

### Analysis of the morphology and stability of amyloid degradation products formed from various proteins after CTSB treatment

**Fig. 2:**
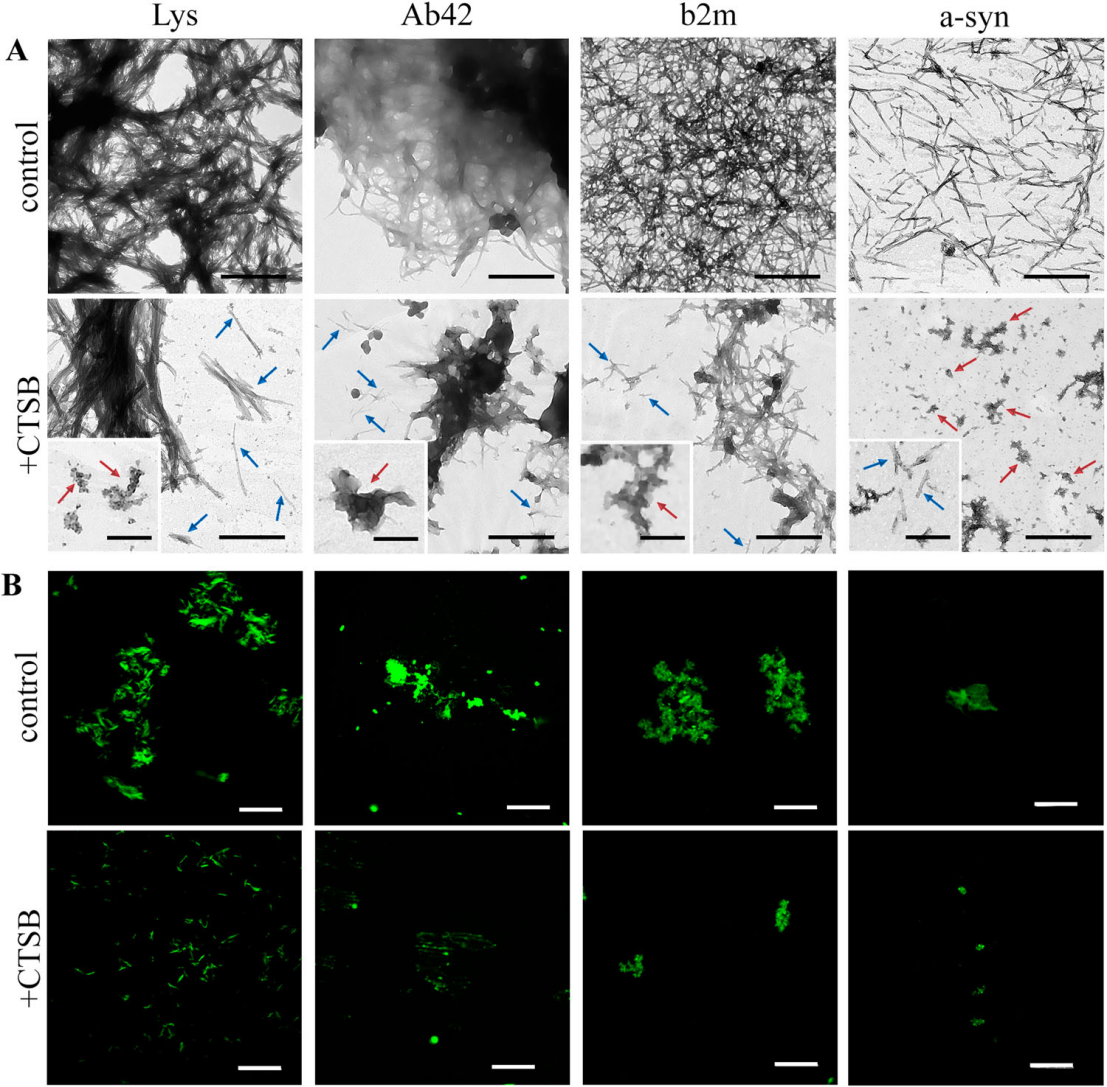
Visualization of amyloid fibrils and their degradation products. Data are presented for amyloids formed from lysozyme (Lys), abeta peptide (1-42) (Ab42), beta-2-microglobulin (b2m), and alpha-synuclein (a-syn) (indicated above the panels) before (control) and 24 h after (+CTSB) CTSB action. **A** Transmission electron microscopy images of the most characteristic morphology of objects in the samples. Scale bars are 0.5 μm. The *Insets* show images of minor protein fractions in the samples. Scale bars are 0.2 μm. The arrows indicate two different types of degradation products: amyloid fragments with intact fibrillar morphology (blue arrows) and aggregates with disrupted morphology (red arrows). **B** Confocal microscopy images of the samples in the presence of ThT. Scale bars are 15 μm.

The degradation products of amyloids formed from lysozyme, abeta peptide (1-42), alpha-synuclein, and beta-2-microglobulin, after 24 h of their incubation in the presence of CTSB, were visualized using transmission electron microscopy (TEM, Fig. 2A). To assess changes in the size of large fibrillary clusters, confocal laser scanning microscopy was used in the presence of the amyloid-specific probe thioflavin T (Fig. 2B). Noted that intact alpha-synuclein amyloids were poorly detectable by confocal laser scanning microscopy, suggesting very few large fibrillar clusters in this sample. It was found that CTSB treatment caused the destruction of all studied amyloids. We observed a noticeable decrease in the size of amyloid clusters (Fig. S1C) and fragmentation of fibrils (shown by blue arrows) and disruption of the ordered morphology of fibrils (shown by red arrows).

**Fig. 3:**
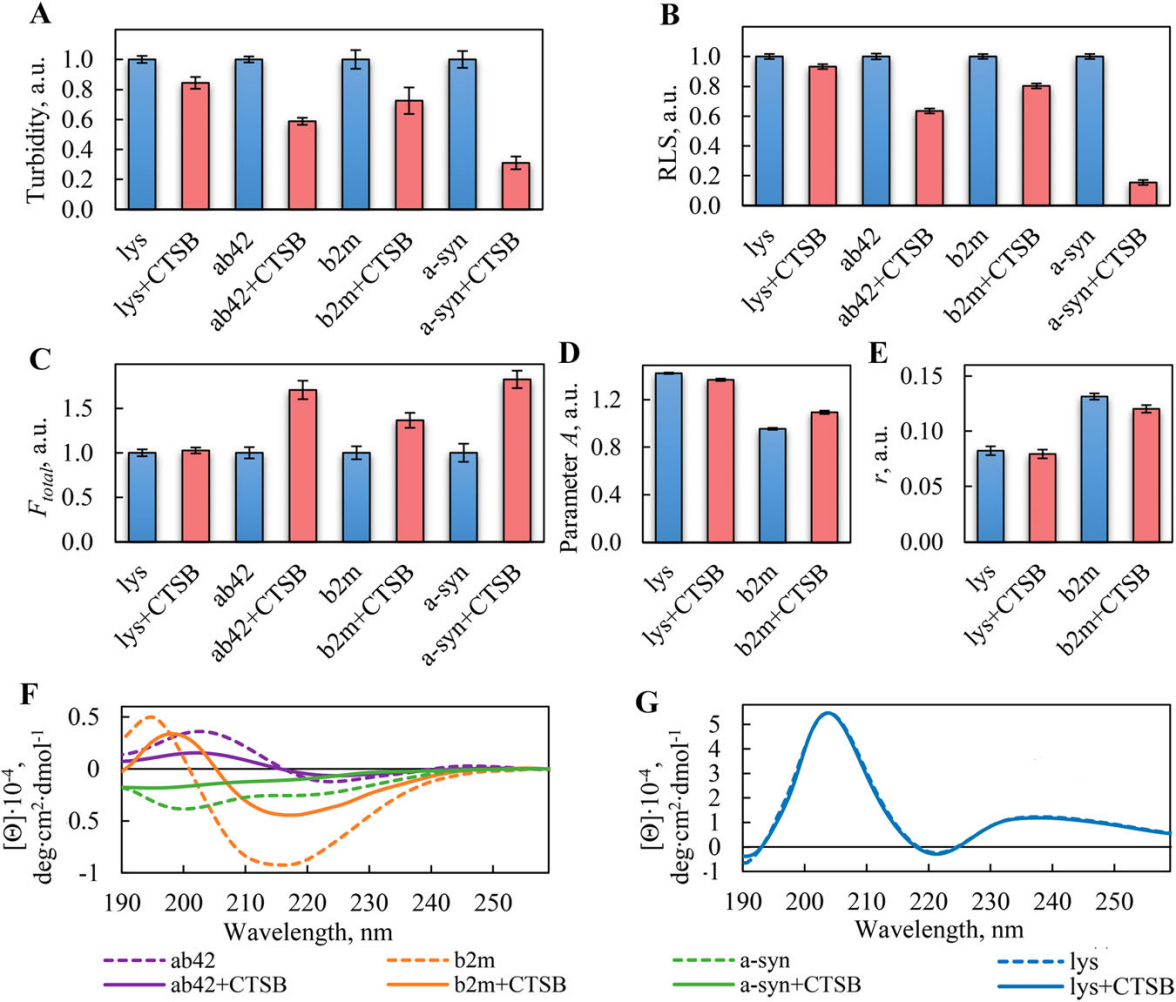
Characterization of the structure and properties of amyloid fibrils and their degradation products. Data are presented for amyloids formed from lysozyme (Lys), abeta peptide (1-42) (Ab42), beta-2-microglobulin (b2m), and alpha-synuclein (a-syn) before (blue bars) and 24 h after (red bars) CTSB action. (**A**) Turbidity, (**B**) Rayleigh light scattering (RLS), (**C**) integrated intensity of intrinsic UV fluorescence, *λex* = 280 nm for abeta peptide (1-42) and alpha-synuclein amyloids, *λex* = 295 nm for lysozyme and beta-2-microglobulin amyloids, (**D**) parameter *A*, *λex* = 295 nm, (**E**) fluorescence anisotropy (*r*), *λex* = 295 nm, *λem* = 365 nm. In (**A**–**C**), values for samples of amyloids with CTSB are normalized to the values for samples of the same intact amyloids. Data are mean ± SD (n = 5). (**F**, **G**) Far-UV CD spectra of amyloid fibrils before (Lys, Ab42, b2m, a-syn) and 24 h after (Lys + CTSB, Ab42 + CTSB, b2m + CTSB, a-syn + CTSB) enzyme action.

We also measured the turbidity and Rayleigh light scattering (RLS) of the samples (Fig. 3A, B). A decrease in these parameters as a result of protease action was observed for all amyloid fibrils, confirming the decrease in the quantity/size of the studied aggregates after treatment. However, the efficiency of amyloid degradation was found to vary. The most pronounced reduction in turbidity and RLS was observed in the case of amyloid fibrils formed from alpha-synuclein (by 70-85%), and the least in the case of amyloid fibrils formed from lysozyme (7–15%).

To confirm the assumption of structural transformations of amyloids in the presence of CTSB, inferred from TEM results, intrinsic UV fluorescence spectra were determined and the value of the integral fluorescence intensity of the samples was calculated (Fig. 3C). Additionally, for fibrils formed from tryptophan-containing proteins, values of fluorescence anisotropy and the parameter *A* (a characteristic defined as the ratio of fluorescence intensity at registration wavelengths of 320 and 365 nm, sensitive to the microenvironment properties of tryptophan residues) were also assessed (Fig. 3D, E). Notably, the intensity of intrinsic UV fluorescence increased in samples with amyloids formed from abeta peptide (1-42), alpha-synuclein, and beta-2-microglobulin after enzyme treatment, indicating a loosening of the protein environment (and the removal of its quenching effect on fluorescence) of aromatic residues. Concurrently, there was also detected a change in parameter *A* for amyloids formed from tryptophan-containing proteins: an increase in this characteristic for beta-2-microglobulin amyloids and a slight decrease for lysozyme amyloids, as well as a decrease in fluorescence anisotropy for beta-2-microglobulin amyloids. Collectively, these results indicate a change in the protein environment of residues contributing to the intrinsic fluorescence of amyloids, which is consistent with the hypothesis of fibril structure disruption upon protease action.

Measuring the circular dichroism (CD) spectra of samples in the far UV region allowed for the assessment of changes in the structural properties of aggregates under the action of CTSB (Fig. 3F, G). All fibrils analyzed in current study display a typical negative band at about 217–225 nm, depending on the fibril morphology, confirming their enrichment in beta-sheet content. For most amyloids, we observed a change in the shape and intensity of the CD spectra after CTSB treatment (Fig. 3F). In particular, after protease action on fibrils of abeta peptide (1-42), alpha-synuclein, and beta-2-microglobulin, the minimum around 220 nm, characteristic of the beta-sheet structure [66] became less pronounced. This confirms the assumption of a decrease in the orderliness of the structure of amyloid fragments, made based on TEM data. However, we did not observe a noticeable change in the CD spectra of lysozyme amyloids after enzyme treatment (Fig. 3G). It can be assumed that changes in the structure of these amyloids cannot be detected by the CD spectroscopy method, both because of their negligibility (which is consistent with changes in intrinsic fluorescence characteristics).

### Studying the interaction of fibrils and their degradation products with the amyloid-specific probe thioflavin T

Along with fibrillary morphology and high content of beta-sheet structure [67], the presence of amyloids in the sample is confirmed by the tinctorial properties evaluated by binding of amyloid-specific probe thioflavin T (ThT) [68]. So, this dye can be effectively used for analyzing fibrils degradation processes [69–72]. The use of ThT for this task is rationalized because the dye specifically incorporates into the grooves formed by the side chains of amino acids of the beta-strands of the fibril, along the long axis of its fiber perpendicular to the beta-sheets (4-5 stacked beta-strands are required for binding 1 molecule of ThT) [73, 74]. The dye does not interact with monomeric proteins in globular, unfolded, or partially folded states, with oligomers, or with amorphous protein aggregates. That means the interaction of ThT with the beta-sheet structure of amyloids is highly specific, and therefore, information about the quantity of bound dye molecules can allow for the assessment of changes in the number of beta-strands in amyloid fibers during their degradation.

**Fig. 4:**
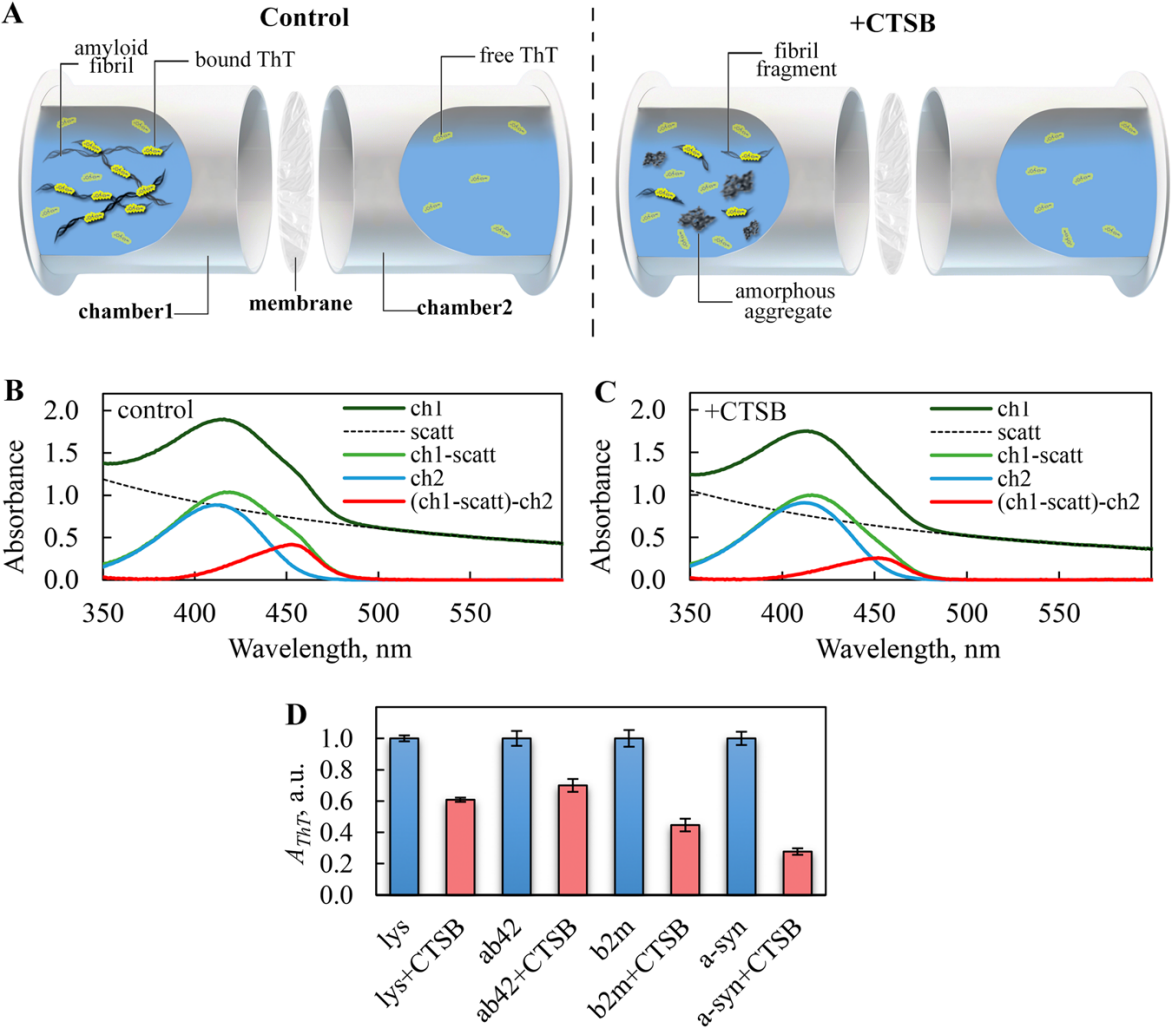
Analysis of the interaction of amyloids and their degradation products with thioflavin T. **A** Equilibrium microdialysis device consisting of two chambers (chamber 1 and chamber 2) of equal volume, separated by a membrane permeable to the fluorescent probe thioflavin T (ThT) but impermeable to protein aggregates and oligomers. In the experiments, amyloids before treatment (control, left) or amyloid degradation products after CTSB action (+CTSB, right) were placed in chamber 1, and a dye solution in the same solvent was placed in the chamber 2. Measurements were taken after the system reached equilibrium and free dye concentration in the chambers became equal (shown at the **A**). Determination of the absorption spectrum of ThT bound to lysozyme amyloid fibrils (**B**) before and (**C**) after CTSB treatment, using samples prepared by equilibrium microdialysis. After reaching equilibrium, the absorption spectrum in chamber 2 (ch2) represented the absorption spectrum of free ThT, while the absorption spectrum of the solution in the chamber 1 (ch1) represented the total absorption spectrum of free ThT and dye bound to fibrils (against a background of “apparent absorption” caused by fibrils light scattering (scatt), which was accounted by a standard procedure (ch1-scatt)). This approach allowed us to obtain solutions of the sample and the reference, whose difference spectrum (ch1-scatt)-ch2) was determined by absorption spectroscopy. This spectrum represents the absorption spectrum of ThT incorporated into the lysozyme fibrils in samples before or after CTSB addition. Similar experiments were performed for all amyloids tested. **D** Absorbance (at the spectral maximum) of ThT (*A*_*ThT*_) bound to fibrils formed from lysozyme (Lys), abeta peptide (1-42) (Ab42), beta-2-microglobulin (b2m), and alpha-synuclein (a-syn) before (blue bars) and after (red bars) CTSB action, determined using samples prepared by equilibrium microdialysis. Values for samples of amyloids with CTSB are normalized to the values for samples of the same intact amyloids. Data are mean ± SD (*n* = 5).

Accurate estimation of the amount (concentration) of ThT bound to fibrils requires the separation of the absorption spectra of the free and fibril-bound dye fractions present in the sample. To address this task, we used a special approach, based on preparing samples via equilibrium microdialysis [75, 76] (Fig. 4A). The essence of this approach lies in introducing amyloids (or their degradation products) suspension into one chamber of the equilibrium microdialysis device and ThT solution into another. Since the chambers are separated by a membrane permeable to the dye but impermeable to the fibrils, after equilibration the system the concentration of free ThT molecules in the chambers becomes equal. However, the total concentration of the dye in the chamber with fibrils (or their degradation products) exceeds the dye concentration in the second chamber by the concentration of ThT molecules bound to undegraded amyloid fibers or their fragments with intact structure (Fig. 4A). Thus, the differential absorption spectrum of the dye in these chambers (adjusted for light scattering of fibrils) represents the spectrum of the dye bound to fibrils, and its amplitude decreases upon amyloid degradation (shown for lysozyme amyloids in Fig. 4B, C).

The reduction in absorption (and thus, concentration) of ThT molecules bound to the studied amyloids before and after their degradation, indicating a decrease in the content of ordered beta-strands in the amyloid degradation products, is presented in Fig. 4D. The most significant decrease in ThT absorption was observed in the case of amyloids formed from alpha-synuclein (over 70%), while the least significant was in the case of amyloids formed from lysozyme and abeta peptide (1-42) (30-40%), confirming the varying efficiency of their degradation.

### Analysis of the impact of amyloids before and after their degradation on the viability of immortalized and primary cell lines

The assessment of the impact on cells of amyloid degradation products after CTSB treatment was conducted using a colorimetric MTT assay. To obtain a comprehensive picture of the cytotoxicity of the studied amyloids and their degradation products during the experiments, several human cell lines were used. Specifically, the study subjects were human embryonic kidney cell lines (Hek-293), epithelioid carcinoma of the cervix cell lines (HeLa TK-), glioblastoma (T98G), and dermal fibroblasts (DF1). The cells were incubated in the presence of amyloids and their degradation products for 24 h.

**Fig. 5:**
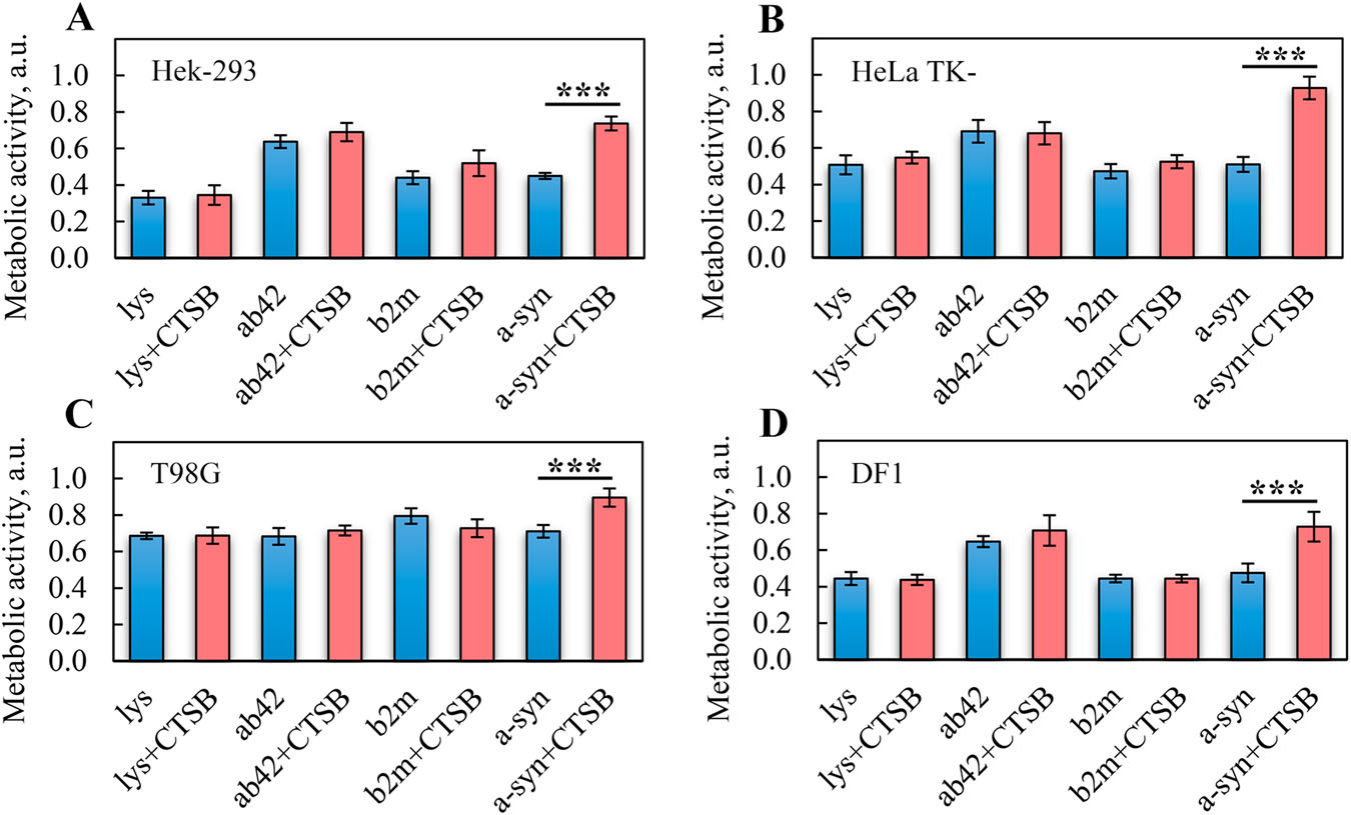
Cytotoxicity of amyloids and their degradation products. Relative metabolic activity of (**A**) human embryonic kidney (Hek-293), (**B**) epithelioid cervix carcinoma (HeLa TK-), (**C**) glioblastoma (T98G), and (**D**) dermal fibroblast (DF1) cell lines after 24 h of exposure to mature amyloids formed from lysozyme (Lys), abeta peptide (1-42) (Ab42), beta-2-microglobulin (b2m), and alpha-synuclein (a-syn) (blue bars) and their degradation products (red bars) generated by the proteolytic enzyme CTSB (Lys + CTSB, Ab42 + CTSB, b2m + CTSB, a-syn + CTSB). In experiments with lysozyme and beta-2-microglobulin amyloids values are normalized to the metabolic activity of control cells to which the target proteins were added in the same buffer and at the same concentration as their corresponding fibrils. In experiments with abeta peptide (1-42) and alpha-synuclein, the cells treated with experimental buffer solution were used as controls. Data are illustrated as the mean (±SD). An unpaired t-test was performed to validate the statistical significance. ***p < 0.0001.

The results of our studies showed (Fig. 5) that amyloids formed from abeta peptide (1-42), alpha-synuclein, lysozyme, and beta-2-microglobulin reduced the metabolic activity of all studied cell lines, which can be considered as a quantitative assessment of amyloid-induced stress in cell culture. The patterns observed in tumor cells (changes in cytotoxicity of amyloids before and after their degradation) were shown to be well reproducible in the primary line of dermal fibroblasts (Fig. 5D).

Our findings indicate a general level of cell line sensitivity to stress induced by extracellular amyloid aggregates. It turned out that the viability of cells in the presence of fibrils was determined not only by amyloid properties but also by the characteristics of the cell lines. For instance, the glioblastoma line (T98G) demonstrated the least sensitivity to all studied amyloids and their degradation products, with the metabolic activity of its cells decreasing by about 30% compared to control cells under the action of all studied amyloids (Fig. 5C). The highest sensitivity was found in human embryonic kidney cells (Hek-293), whose metabolic activity in the presence of some amyloids decreased by almost 70% compared to control samples (Fig. 5A). Interestingly, the sensitivity of the primary dermal fibroblast line (DF1) was comparable to the sensitivity of the Hek-293 line (Fig. 5D), both regarding amyloids associated with neurodegenerative diseases and amyloids leading to local and systemic forms of amyloidosis.

It was found that the impact of intact fibrils formed from lysozyme, beta-2-microglobulin, and abeta peptide (1-42) on the viability of cell lines was comparable to that of the degradation products of these amyloids (Fig. 5). Meanwhile, the cytotoxicity of fibrils formed from alpha-synuclein significantly decreased after their degradation by the action of CTSB. This effect was observed for all studied cell lines. In particular, when cells of the HeLa TK- line were exposed to the degradation products of amyloids formed from alpha-synuclein, their viability was almost completely restored to the level of intact cells (Fig. 5B).

## Discussion

### Factors determining the mechanism and efficiency of CTSB action on amyloid fibrils

The experimental data obtained allowed us to establish that CTSB-induced proteolysis of proteins in the composition of fibrils leads to the fragmentation of these aggregates (Figs. 1, 2). This means that the action of the enzyme on amyloid-forming proteins results in the disruption of hydrogen bonds between the beta-strands of the amyloid fiber. However, the mechanism of amyloid degradation under the action of CTSB appears to be dual. Along with fragmentation, the loss of the ordered structure of fibril clusters and fragments was observed (Figs. 1, 2), implying that CTSB can also causes destabilization of intramolecular contacts (hydrophobic, ionic, and/or covalent). None of the fibrils studied appeared to undergo complete dissociation into monomeric subunits. The degradation products after CTSB action predominantly consisted of fragments of the amyloid fiber varying in size with intact beta-strands, and protein aggregates of different sizes with a “loosened” tertiary structure and a less defined secondary structure. No reassembly of the fibrils was observed (as is the case with some other factors that disrupt amyloids) following termination of proteolytic enzyme action. Thus, CTSB-induced degradation of amyloids is irreversible.

It was found that despite a similar dual mechanism of enzyme influence on different amyloid fibrils, the effectiveness of this action noticeably varies. The most efficient degradation was observed in the case of aggregates formed from alpha-synuclein (Figs. 2–4). These amyloids predominantly consist of thin individual fibers which, in rare cases, are capable of interacting with each other, forming a small number of «loose» clusters. Meanwhile, amyloids formed from lysozyme and abeta peptide (1-42), which have a larger diameter and/or are assembled into large number of dense clusters, proved to be the most resistant to the effect and did not change their size and morphology as significantly. This means that the efficacy of the fibrils degrading likely depends on the accessibility of CTSB binding sites. In particular, the enzyme cannot act on protein sites located within a dense/thick amyloid fiber and on proteins located deep within dense fibrillar clusters, resulting only in partial degradation of such amyloids at the periphery or in the least shielded areas from the action.

Thus, considering the results obtained, it can be hypothesized that in the case of CTSB application in anti-amyloid therapy, the enzyme will exhibit a universal dual mechanism of action on fibrils regardless of the pathology and type of amyloids causing it. However, the properties of these amyloids (structure, size, ability to form dense clusters, etc.) will largely determine the effectiveness of the degrading action as well as predominant type of degradation products.

### The capacity of CTSB to reduce the cytotoxicity of amyloid fibrils

A survey of the available literature indicates that exposure to a variety of exogenous factors, particularly proteolytic enzymes such as trypsin and alpha-B-crystallin, can result in elevated amyloid toxicity towards cells [70, 71, 77]. This heightened toxicity is hypothesized to arise from either an increase in the affinity of amyloids degradation products for the cell membrane, leading to its excessive destabilization, or conversely, addition stabilization. However, our evaluation of the cytotoxic effects of amyloid degradation products formed from various proteins (lysozyme, beta-2-microglobulin, and abeta peptide (1-42), associated with lysozyme and hemodialysis amyloidosis as well as Alzheimer’s disease, respectively) suggests that CTSB can degrade amyloid fibrils without forming low-molecular-weight products with higher cytotoxicity (Fig. 5). Moreover, this enzyme not only reduces the size of aggregates (thus facilitating their further degradation) but, in some cases, also alleviates cell stress induced by amyloids. Specifically, for all cell lines examined (T98G, Hek-293, DF1, and Hela), the toxicity of degradation products of alpha-synuclein amyloids, associated with Parkinson’s disease, was significantly lower compared to intact fibrils (Fig. 5). This is consistent with literature data showing the positive impact of this enzyme on the pathogenesis of amyloidoses [40, 42, 50]. We believe that the significant reduction in cytotoxicity specifically for alpha-synuclein fibrils is attributed to the most efficient degradation of these amyloids, leading both to effective fragmentation and destruction of the ordered fibrillar structure. As previously noted, this is likely related to the thin structure and low tendency of these fibrils to form dense clusters, resulting in higher accessibility of proteolytic cleavage sites. Therefore, CTSB and other factors with similar mechanisms of action can be considered potential components of anti-amyloid therapeutic agents, leading to effective degradation and reduced cytotoxicity of degradation products, provided that amyloid plaques are pre-declustered.

### “Pitfalls” and advantages of using CTSB as an anti-amyloid agent

Despite our findings indicating no increase and, in some cases, a significant decrease, in amyloid fibril cytotoxicity following CTSB exposure, suggesting effective degradation, the observed mechanism of degradation suggests potential “side effects” of such treatment. We demonstrated that one of the degradation products of amyloids under CTSB action are fibril fragments with a structure identical to intact amyloids. These short fibrils and oligomers can serve as “seeds” for the formation of new amyloid fibrils (i.e., they can accelerate fibrillogenesis by increasing the number of “free sticky ends”) and can also rapidly spread between cells. For instance, it has been previously shown that the degradation of amyloids into fibrillar fragments can be a critical step in the propagation of prions and prion-like aggregates in yeast [78, 79]. Similar mechanisms are likely to occur in mammalian cells during amyloid degradation [80]. It can be speculated that such a CTSB-induced mechanism of alpha-synuclein amyloid degradation accounts for the increased nucleation activity and induction of intracellular alpha-synuclein aggregate formation observed in studies [48, 51]. However, it is important to note that other studies emphasize the positive effect of CTSB on the pathogenesis of amyloid-related diseases [40, 42, 50].

According to our results, we hypothesize that the conflicting data on the role of CTSB in amyloidoses may be attributed to the destruction mechanism duality. We consider by this enzyme’s ability to trigger two different processes: 1) accelerating fibrillogenesis through fibril fragmentation (a negative effect), and 2) reducing the cytotoxic and seeding activity of amyloids by “disordering” their structure (a positive effect). Thus, the observed change in amyloid pathogenicity will largely depend on which of these processes prevails. If amyloids are highly clustered (as observed in the case of lysozyme amyloids in our experiment) and resistant to CTSB degradation, their fragmentation will probably be the predominant outcome. This process would generate smaller clusters without substantially impacting cytotoxicity but would, however, facilitate a more rapid rate of amyloid formation and propagation between cells and tissues. In contrast, when amyloids present as thin, individual fibers, that do not form dense clusters, as observed with the alpha-synuclein amyloids in our study, or have undergone a preliminary declustering process, the action of CTSB is expected be more effective. This enhanced action would lead not only to a reduction in aggregate size but also to a disruption of the fibrillar structure itself. In this scenario, degradation products will not act as seeds for fibrillogenesis and will have low cytotoxicity for cells. Thus, our results suggest that CTSB could become a component of effective and safe anti-amyloid drugs, in combination with other factors that enhance the accessibility of proteolytic sites and reduce the stability of amyloid clusters.

## Conclusions

**Fig. 6:**
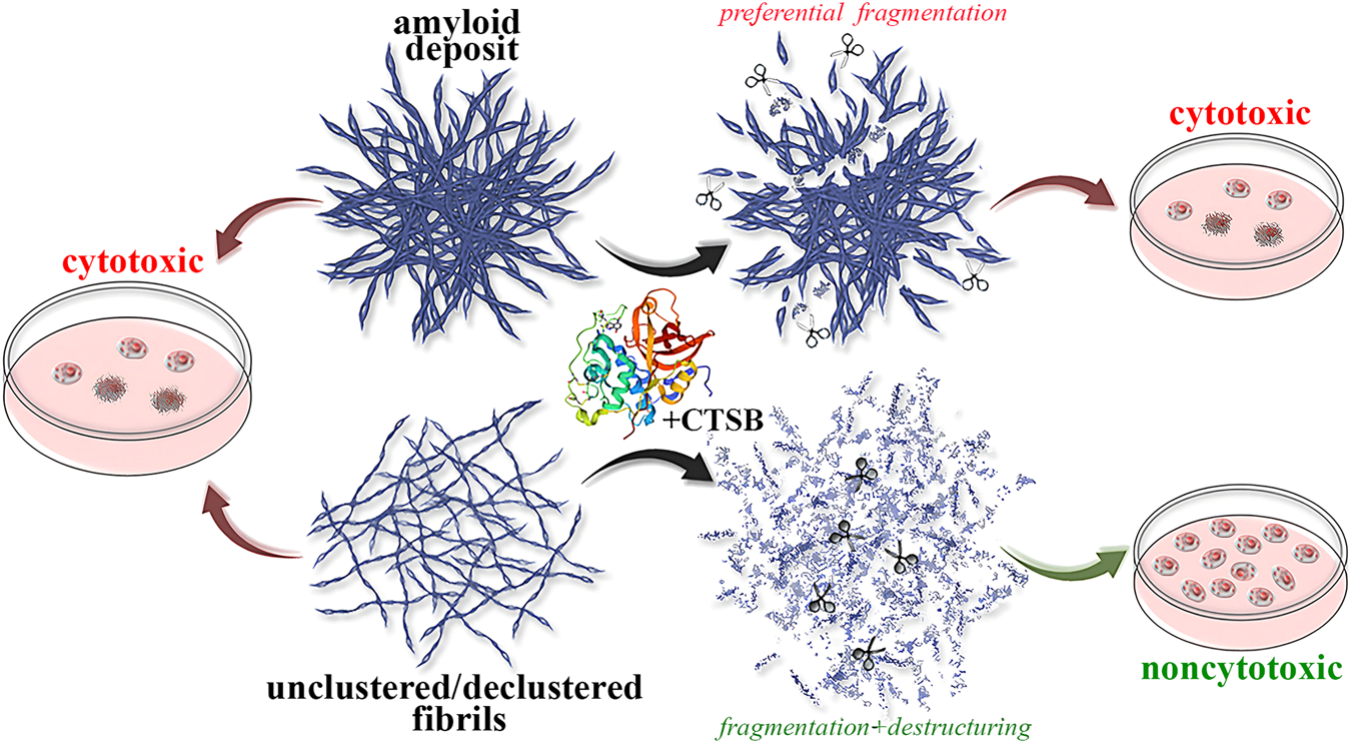
Scheme illustrating the implications of the CTSB effect on amyloid fibrils with different clustering tendencies. Our study shows that CTSB induces both disruption of the structure and fragmentation of amyloids. The change in amyloid cytotoxicity depends on which process prevails. For highly clustered amyloids resistant to CTSB degradation, fragmentation is likely the predominant outcome (top). This process generates smaller clusters and fragments with intact fibrillar structure without significantly impacting cytotoxicity. In contrast, for amyloids present as thin individual fibers or those that have undergone preliminary declustering, the action of CTSB is expected to be more effective (bottom). This enhanced action leads to a reduction in aggregate size and disruption of the fibrillar structure itself. In this scenario, degradation products have reduced toxicity for cells and do not act as seeds for fibrillogenesis like non-disrupted fragments. Thus, CTSB could be a key component in safe, effective anti-amyloid drugs, combined with factors enhancing proteolytic site accessibility and cluster stability reduction.

This study focused on evaluating the degradation potential of CTSB on amyloid fibrils with varying clustering tendencies, associated with Alzheimer’s and Parkinson’s diseases, hemodialysis, and lysozyme amyloidosis. We demonstrated, for the first time, the ability of CTSB to induce both the disruption of amyloid structures and their fragmentation, with corresponding changes in amyloid cytotoxicity depending on which process predominated (Fig. 6). These findings significantly advance our understanding of how endogenous CTSB may regulate amyloid accumulation at the molecular level across diverse neuropathologies. Furthermore, our results indicate the potential of CTSB as a therapeutic component in anti-amyloid drug strategies, particularly when used in combination with agents that enhance the accessibility of proteolytic sites within amyloid aggregates and destabilize dense fibrillar clusters.

## Materials and methods

### Materials

Fluorescent dye thioflavin T (ThT) “UltraPure Grade” from AnaSpec (USA), 1,1,1,3,3,3-Hexafluoro-2-propanol (HFIP), 3-(4,5-dimethylthiazol-2-yl)-2,5-diphenyltetrazolium bromide (MTT), and buffer components from Sigma (USA) were used without additional purification. Reagents for cell cultivation, including DMEM medium (glucose 4.5 g/l), fetal bovine serum (FBS), and 0.25% Trypsin-EDTA were acquired from Gibco (Thermo Fisher Scientific, USA). Culture flasks and 96-well plates (flat bottom) were purchased from Corning (USA).

The recombinant human beta-2-microglobulin was expressed, purified using the protocol [81], human abeta peptide (1-42) was acquired from GL Biochem (China), lysozyme from chicken egg white was acquired from Sigma-Aldrich (USA), cathepsin B was acquired from Merck (Germany).

The recombinant human alpha-synuclein was expressed in E. coli BL21(*DE3*) star cells transformed with the pT7-7 plasmid encoding for the target protein. Cells were cultivated in Terrific Broth (TB) medium supplied with 100 μg/ml of ampicillin. Expression of the target protein was induced by 0.5 mM of isopropyl beta-D-1-thiogalactopyranoside (IPTG) for 20 h at 18 °C. The cells harvested by centrifugation (5000 rpm, 30 min, 4 °C) were frozen/thawed, resuspended in 10 mM TrisHCl buffer, pH 8.0, 1 mM EDTA, 1 mM protease inhibitor phenylmethylsulfonyl fluoride (PMSF) and undergone to hyperosmotic shock [82]. The fraction of target protein was collected as supernatant by centrifugation for 30 min at 5000 rpm. Further purification of alpha-synuclein was achieved by thermo-precipitation of impurity proteins (boiling at 100 °C for 15 min, cooling at 4 °C for 5 min, followed by centrifugation at 12000 rpm for 30 min at 4 °C). Next, the target protein was precipitated by 40% ammonium sulfate. Final purification of alpha-synuclein was carried out with Mono-Q 5/50 GL (GE Healthcare, Danderyd, Sweden). The purified alpha-synuclein were dialyzed against 10 mM TrisHCl buffer, pH7.6, 25мМ NaCl and stored at −80 °C.

### Amyloid fibrils preparation and their treatment with CTSB

Abeta peptide (1-42) and alpha-synuclein in final concentration 1 mg/ml were solubilized in 50% organic solvent 1,1,1,3,3,3-Hexafluoro-2-propanol (HFIP) and incubated for 7 days [83–85]. Following this period, the HFIP was slowly removed through evaporation with a stream of nitrogen. Then volume of the sample was adjusted with distilled water to the initial one, and the samples were incubated for an additional week. Lysozyme in concentration 2 mg/ml was incubated in 100 mM KH_2_PO_4_-NaOH buffer (pH 7) in the presence of 3 М guanidine hydrochloride (GdnHCl) at 57 °C for 2 days [86]. For beta-2-microglobulin amyloid fibrils preparation, isolated and purified protein in concentration 1 mg/ml was incubated in 100 mM Gly-HCl buffer (pH 2) at 37 °C for 7 days [81]. All amyloid fibrils were prepared at constant agitation (500 rpm).

The presence of amyloid fibrils in the samples was confirmed by transmission electron microscopy. Following the formation of mature amyloid fibrils for performing experiments protein aggregates were dialyzed against Milli-Q water. The fibril stability and the retention of their characteristics for at least the time during which the experiment with cathepsin B was carried out were controlled.

Pre-activated in 50 mM sodium acetate buffer (pH 5)/1 mM ethylenediaminetetraacetic acid (EDTA) with 1 mM dithiothreitol (DTT) according to the manufacturing protocol, cathepsin B was added to the amyloid fibrils in a 1/50 ratio. To inactivate cathepsin B samples were incubated at 57 °C for 10 min [65]. Fibrils obtained using highly reproducible protocols were subjected to protease treatment multiple times with repeatable effects. All samples were included in the analysis.

### Transmission electron microscopy

A transmission electron microscope Libra 120 (Carl Zeiss, Germany) was used to produce the micrographs of amyloid fibrils and products of their degradation. Samples were put on the copper grids coated with formvar/carbon films (Electron Microscopy Sciences, USA) and stained by a 1% aqueous solution of uranyl acetate. The length of amyloid fibrils and their fragments was estimated by the ImageJ program.

### Confocal microscopy

Confocal laser scanning microscope Olympus FV 3000 (Olympus, Japan) and oil immersion objective with a 60× magnification, the numerical aperture (NA) of 1.42, and laser with excitation line of 405 nm were applied to visualize the ThT-stained amyloid samples. The size of amyloid fibril clusters was estimated as the area in the ImageJ program.

### Spectral measurements

A U-3900H spectrophotometer (Hitachi, Japan) was applied to collect the absorption spectra of the samples. The absorption spectra of amyloid fibrils and mixtures of fibrils with ThT were corrected by the light scattering according to the standard procedure [87]. The size of aggregates was characterized by turbidity recorded at 530 nm.

Fluorescence spectra of the samples were obtained through the Cary Eclipse spectrofluorimeter (Varian, Australia). Intrinsic fluorescence of tryptophan and tyrosine residues of amyloid-forming proteins was recorded using 295 and 280 nm excitation wavelengths. Using parameter *A*, that is ratio of the fluorescence intensities at the emission wavelengths of 320 and 365 nm, the change of the position and shape of the fibrils fluorescence spectra was analyzed. Rayleigh light scattering (RLS) was determined using the same wavelength of excitation and registration (530 nm). The anisotropy of tryptophan fluorescence was calculated using the vertical and horizontal components of the fluorescence intensity excited by vertically polarized light and recorded at the wavelength of 365 nm. ThT fluorescence was excited at 440 nm. All recorded values of fluorescence intensity were adjusted for the primary inner filter effect [88].

For the measurement of circular dichroism (CD) spectra, a J-810 spectropolarimeter (Jasco, Japan) was used. The far-UV CD spectra were recorded in the range of 190–260 nm with a step of 0.2 nm and standard instrument sensitivity using a 1 mm path length cell. To prevent the formation of optically active ozone, the measurements were carried out under continuous nitrogen supply, which ensured efficient removal of oxygen from the optical path of the instrument. Aqueous solutions (Milli-Q water) of amyloid fibrils were used to record optical activity. Milli-Q water is optically transparent up to 180 nm at 1 mm optical path. To achieve an optimal signal-to-noise ratio in circular dichroism measurements and to reduce the light scattering of the samples, we used a relatively low sample concentration (0.15–0.2 mg/mL). Spectra were recorded at a scanning speed of 10 nm/min and a response time of 8 s to increase the signal-to-noise ratio as well. Three scans of CD spectra were averaged and corrected for baseline drift using the signal of Milli-Q water.

### Equilibrium microdialysis

The tested solutions of ThT with amyloid fibrils were prepared by equilibrium microdialysis using a Harvard Apparatus/Amika (USA) device [75, 76]. Equilibrium microdialysis was performed at fibrils (or their degradation products) and ThT concentrations of 0.5 mg/ml and 30 μM, respectively. The concentration of free ThT was quantified using molar extinction coefficient at 412 nm of 31600 M^−1 ^cm^−1^ [89].

### MTT assay

Human embryonic kidney (Hek-293), cervical epithelioid carcinoma (HeLa TK-), glioblastoma (T98G) and dermal fibroblast (DF1) cells were used. Cells were obtained from the shared research facility “Vertebrate cell culture collection”. All cell lines were authenticated by STR profiling and were checked for mycoplasma infections. The metabolic activity was evaluated in 24 h after the addition of the analyzed amyloids and their degradation products at 0.003 mg/ml concentration, using an MTT inhibition assay according to a standard protocol as described earlier [90, 91].

### Pseudo-native sodium dodecyl sulfate (SDS) gel electrophoresis

The size of amyloid fibril degradation products after CTSB action was analyzed by sodium dodecyl-sulfate polyacrylamide gel electrophoresis (SDS-PAGE) analysis on 17% and 8% polyacrylamide gel (0.375 M Tris HCl, pH 8.8, 0.1% SDS). Samples were loaded on the gel in a buffer containing 0.0625 M Tris HCl, pH 6.8, 1% SDS, 10% glycerol, and 0.002% bromophenol blue without boiling.

### Statistical analysis

All data were obtained in 5 independent biological replicates. GraphPad Prism 10.3.0 (GraphPad, CA, USA) was used for statistical analysis, with the data presented as mean ± standard deviation. All data followed a normal distribution as determined by the Shapiro–Wilk test. The equality of variances between groups was validated by Levene’s test. Statistical analysis of the differences between groups were performed using an unpaired two-tailed *t*-test with a 99% confidence interval. Results were considered statistically significant when *p*-value of less than 0.01.

## Supplementary information

SUPPLEMENTAL MATERIAL

Original SDS-PAGE gels

## Data Availability

All analyzed and generated data during current study are included in the article and Supplementary information. Further information is available from the corresponding author on reasonable request.
