# Supplementary material for: Cathepsin B prevents cell death by fragmentation and destruction of pathological amyloid fibrils
Source: Cell Death Discov. 2025 Feb 15;11:61. doi: 10.1038/s41420-025-02343-w (PMC11830053; doi:10.1038/s41420-025-02343-w)
Supplement: Supplementary file 1 — SUPPLEMENTAL MATERIAL [file 41420_2025_2343_MOESM1_ESM.docx]

**Cathepsin B prevents cell death by fragmentation and destruction of pathological amyloid fibrils**

**Maksim I. Sulatsky^1,†^, Olesya V. Stepanenko^2,†^, Olga V. Stepanenko^2^, Ekaterina V. Mikhailova^2^, Anna I. Sulatskaya^2,^***

^1^ Laboratory of cell morphology, Institute of Cytology Russian Academy of Sciences, 4 Tikhoretsky ave., 194064 St. Petersburg, Russia; [m_sulatsky@mail.ru](mailto:m_sulatsky@mail.ru)

^2^Laboratory of structural dynamics, stability and folding of proteins, Institute of Cytology Russian Academy of Sciences, 4 Tikhoretsky ave., 194064 St. Petersburg, Russia; lvs@incras.ru; sov@incras.ru; 4evamkh@gmail.com; ansul@mail.ru

^†^These authors contributed equally to the work

*****Corresponding author: Anna I. Sulatskaya, Laboratory of structural dynamics, stability and folding of proteins, Institute of Cytology Russian Academy of Sciences, 4 Tikhoretsky ave., 194064 St. Petersburg, ansul@mail.ru


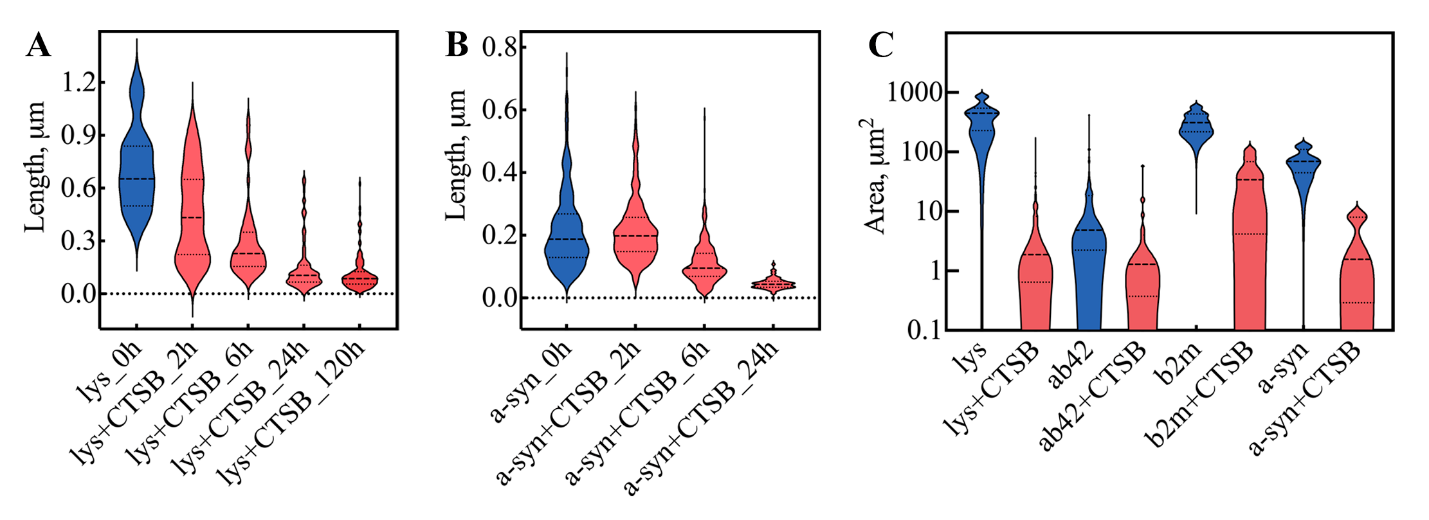


**Figure S1. Estimation of the separate fibril length and amyloid cluster size.** Medians and interquartile ranges are represented by dashed and dotted lines. For amyloid fibrils of each protein, up to 300 objects were analyzed.


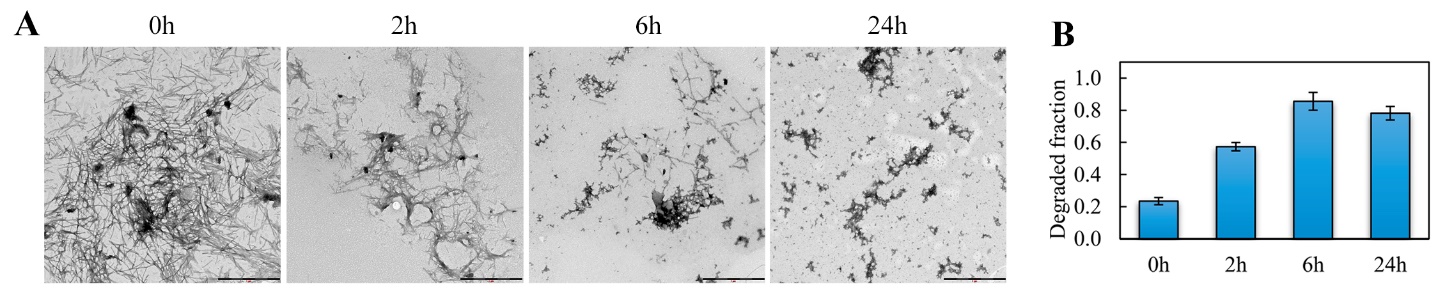


**Figure S2. Dynamics of CTSB-**~~d~~**induced degradation of alpha-synuclein amyloid fibrils.** (A) Visualization of amyloids using transmission electron microscopy before and at different time intervals (2, 6, and 24 h, indicated above the panels) after enzyme addition. Scale bars are 5 μm. (B) Quantification of the degraded fraction in the supernatant using absorption spectroscopy after centrifugation of samples collected at different time intervals (2, 6, 24, and 120 h) after enzyme addition. Values are calculated relative to the concentration of intact amyloid fibrils. Data are mean ± SD (n=5).
