# Supplementary figures and images for: Cathepsin B prevents cell death by fragmentation and destruction of pathological amyloid fibrils
Source: Cell Death Discov. 2025 Feb 15;11:61. doi: 10.1038/s41420-025-02343-w (PMC11830053; doi:10.1038/s41420-025-02343-w)

Figure 1B

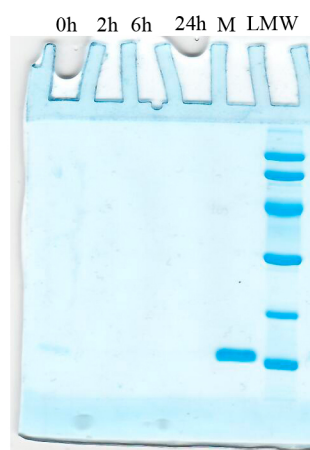

Figure 1C

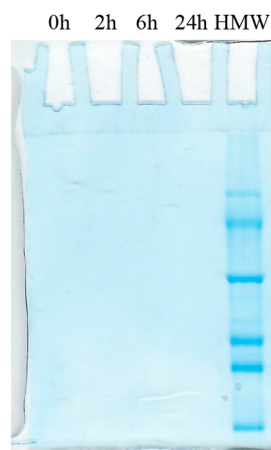

Supplement: Supplementary file 2 — Original SDS-PAGE gels [file 41420_2025_2343_MOESM2_ESM.pdf]
